# *Kursi Wufarikun Ziyabit* Improves the Physiological Changes by Regulating Endoplasmic Reticulum Stress in the Type 2 Diabetes db/db Mice

**DOI:** 10.1155/2021/2100128

**Published:** 2021-08-16

**Authors:** Salamet Edirs, Lan Jiang, XueLei Xin, Haji Akber Aisa

**Affiliations:** ^1^The Key Laboratory of Plant Resources and Chemistry of Arid Zone, Xinjiang Technical Institute of Physics and Chemistry, Chinese Academy of Sciences, 40-1 Beijing Road, Urumqi, Xinjiang 830011, China; ^2^State Key Laboratory Basis of Xinjiang Indigenous Medicinal Plants Resource Utilization, Xinjiang Technical Institute of Physics and Chemistry, Chinese Academy of Sciences, 40-1 Beijing Road, Urumqi, Xinjiang 830011, China

## Abstract

*Kursi Wufarikun Ziyabit* (KWZ) is a classic traditional medicine used for the prevention treatment of diabetes in China. It was widely used as healthcare tea in folk and can prevent and treat type 2 diabetes. However, the underlying mechanism of KWZ in type 2 diabetes has not been investigated extensively. Here, the weekly body weight and blood glucose level of KWZ in type 2 diabetes db/db male mice were observed. After 4 weeks of treatment, the physiological changes and pharmacological effects of KWZ were investigated. The results showed that KWZ can significantly decrease fasting blood glucose and improve glucose tolerance and insulin sensitivity in db/db mice. The serum/liver lipid profiles such as LDL-C, TC, TG, and serum-free fatty acid/Fructosamine levels were decreased, and the serum/liver HDL-C levels were increased. In addition, significant improvement in glucose metabolism enzymes and antioxidant enzymes in experimental mice's livers was observed. Moreover, the expression of GRP78, p-IRE1*α*/IRE1*α*, p-eIF2*α*/eIF2*α*, and XBP1s was decreased. The expression of p-PERK/PERK, p-Akt/Akt, and p-GSK-3*β*/GSK-3*β* was markedly increased. These results suggested that KWZ is effective for type 2 diabetes by improving endoplasmic reticulum (ER) stress in the liver of db/db mice, and it might prevent the damage of insulin Beta cells and alleviate insulin resistance.

## 1. Introduction

Type 2 diabetes mellitus (T2DM) is the most common metabolic disorder disease, and millions of people have been longsuffering the persistent hyperglycemia, hyperinsulinemia, and its numerous complications. As a major public health problem, T2DM is the result of an unfortunate combination of insulin resistance and Beta cell failure [1]. For many years, most subjects have been compensated for by increased insulin secretion in the development of Beta cell function. Earlier studies showed that dysfunction of Beta cell was the crucial factor for the decline in glucose tolerance, particularly in the earliest stages of T2DM [2]. Most T2DM patients have a loss of Beta cell function and apoptosis, but recent studies have shown that the endoplasmic reticulum (ER) response is especially important in secretory cells and can play key roles in insulin resistance [3].

ER is the major site of calcium storage, folding, and maturation of secreted and transmembrane proteins [4]. The most important role of ER is to maintain protein homeostasis. Besides, the ER also controls cholesterol production, lipid biosynthesis, surviving, and death signaling mechanisms in the cell [5]. The loss of ER homeostasis, such as expression of mutant unfolded or misfolded proteins, insufficient ER chaperone levels, calcium content, changes of ATP status, and cholesterol accumulation, leads to ER stress response and induces the diseases such as obesity, diabetes, atherosclerosis, and cancer [6]. However, ER stress response can restore the irregularity of physiological conditions through the activation of the PERK-eIF2*α*-ATF4 pathway, IRE1-XBP1 pathway, and ATF6 pathway [7]. 78-kDa glucose-regulated protein (GRP78) is a major ER chaperone with antiapoptotic properties and a key regulator of the ER stress response, which also plays an important role in regulatory pathways [8]. The activation of IRE1/XBP-1, PERK/eIF2*α*, and ATF6 signaling pathways improves the function of insulin Beta cells through promoting the normal function of ER, such as improving the insulin sensitivity and reducing the fasting blood glucose by reducing the gluconeogenesis [9].

With the rise of diabetes around the world, natural products and their derivatives have been recognized as important sources for new drugs and therapeutic products in the pharmaceutical industry [10]. Natural herbal medicines can protect against many diseases, including diabetes mellitus and its complications through, but not limited to, decreasing oxidative stress, inflammation, and apoptosis [11–13]. *Kursi Wufarikun Ziyabit* (KWZ) is a classic traditional medicine used for the prevention and treatment of diabetes in central Asia, and it was mentioned in Avicenna's “Canon of Medicine” [14]. KWZ consists of two herbs, *Geranium collinum* (*G. collinum*) Steph. ex Willd and *Hypericum scabrum* (*H. scabrum*) Linn., and they were recorded in “Chinese pharmacopeia” [15, 16]. *G. collinum* is rich in polyphenols, flavonoids, organic acid, and terpenoids [17]. *H*. *scabrum* contained a variety of compounds such as tannins, flavonoids, hyperforin derivates, and essential oil [18]. *G. collinum* and *H*. *scabrum* have many biological activities such as antioxidant, anti-inflammatory, antidiarrhea, ulcer healing, decreasing blood sugar, and prevention and treatment of diabetes complications. Recently, many researches have proved their excellent activities on hypoglycemic and antioxidants [19, 20]. We have referred to the above report messages, which previously found the effective part of two herbs, named as *Kursi Wufarikun Ziyabit* (KWZ). Firstly, we optimized the best proportion and extraction conditions, identified the main components, and detected the antidiabetic activities by protein tyrosine phosphatase-1B (PTP-1B) and *α*-glucosidase inhibitors [21]. Then, the hypoglycemic effect of KWZ on L6 rat skeletal muscle cells and its mechanism was investigated, mainly including the glucose consumption, treatment time, and the expression of the major proteins in insulin resistance and ER stress signaling pathways [22]. However, the molecular mechanisms of KWZ are still unknown on db/db T2DM mice. Therefore, we investigated the effect of KWZ on T2DM db/db male mice and its association with the ER stress signaling pathway in liver tissue in the present study.

## 2. Materials and Methods

### 2.1. Materials, Reagents, and Antibodies

All the solvents used for extract were of analytical grade (Baishi Chemical Co. Ltd., Tianjin, P. R. China), and water was double distilled. Metformin hydrochloride tablets were purchased from Harbin Tongyitang Pharmaceutical Co., Ltd (China). Antibodies to GRP78, *ß*-actin, phospho-IER1*α*, phosphor-PERK, phospho-e1F2*α*, XBP1s, IER1*α*, PERK, and e1F2*α* were obtained from Cell Signaling Technology (Danvers, MA, USA). The secondary antibody was purchased from Boster Biological Technology Co., Ltd (China). Electrophoresis reagents, including Bis-Tris gels, running buffer, and poly (vinylidene fluoride) (PVDF) membrane, were obtained from Invitrogen (Carlsbad, CA, USA). ECL was bought from GE Healthcare (UK). Kit of low-density lipoprotein-cholesterol (LDL-C), high-density lipoprotein-cholesterol (HDL-C), blood plasma free fatty acid (FFA), total cholesterol (TC), triacylglyceride (TG), fructosamine, hexokinase (HK), glycogen, catalase (CAT), total Superoxide Dismutase (T-SOD), glutathione peroxidase (GSH-Px), and malondialdehyde (MDA) were purchased from Nanjing Jiancheng Bioengineering Institute (China). Pierce TMT® Mass Tagging Kits and Reagents were purchased from Thermo Science Pierce (Rockford, USA).

### 2.2. Preparation of KWZ for Animal Experimentation

The roots of *G. collinum* and aerial parts of *H. scabrum* were collected from Takob village of the Republic of Tajikistan (38.5357500 N, 68.7790500 E, and 2000 m above sea level, Tajikistan). The plants were identified by Professor Yusuf Nuraliev from Avicena's Institute of Medicine and Pharmacology of the Republic of Tajikistan. Voucher specimens (Barcode: *G*. *collinum* WY01053, *H*. *Scabrum* WY01054) were deposited at the Herbarium of the Key Laboratory of Plant Resources and Chemistry of Arid Zone, Xinjiang Technical Institute of Physics and Chemistry, Chinese Academy of Sciences. The KWZ (the roots of *G. collinum* and aerial parts of *H. Scabrum* were mixed with the ratio of 7 : 3) were prepared according to the article [21]. The dried matter after preparation was powdered, weighed, and packed in zip pack bags, stored at 4°C for further study. The detailed process is as follows: the first root of G*. collinum* and aerial parts of *H. Scabrum* were mixed with the ratio of 7 : 3, placed in a round-bottom flask, and then was extracted (reflux extraction) with 50% ethanol (1 : 20 w/v) for three times, each time for 3 h at 70°C. Then, the extract was concentrated under vacuum to 1.02 g/mL. Concentrated extract (250 mL) was purified with a column of HPD300 macro reticular resin (50 mL). Firstly, it was washed with 150 mL of distilled water and then eluted with 100 mL of 30% ethanol, and after that, it was eluted with 150 mL of 70% ethanol. Thereafter, the eluted parts of 30% ethanol and 70% ethanol were combined, and then they were concentrated and dried using freeze-drying (FDU-2100; Eyela, Tokyo, Japan) at −80°C for 36 h. The dried matter was powdered, weighed, and packed in zip pack bags, stored at 4°C for further study.

### 2.3. Acute Toxicity Test

KWZ was tested for acute toxicity in KM (Kunming) mice (7–8 weeks old) before the beginning of chronic hypoglycemic experiment. The lethal dosage was found in a pretest in the acute toxicity study. All experiment procedures (including study protocol) were approved by the Changzhou Cavens Lab Animal Co., Ltd (Changzhou Cavens Ethics Committee on Animal Experimentation, China, production certificate no: SCXK2016-0010). The experimental animals were housed in the SPF level animal room with 22–24°C temperature, 45–80% humidity, and 12 h light/dark cycle. Three days of quarantine before the experiment, after general behavior observation, select the animals that meet the requirements for the experiment. A total of 66 mice were divided into 11 groups based on their weight with 6 animals per group containing 3 male and 3 female mice. KWZ was tested at 10 dosages: 0, 300, 464, 714, 1100, 1690, 2600, 4000, 4200, and 4500 mg/kg, and oral gavage twice in a volume of 0.2 ml/10 g. The first group was the solvent control group, and the solvent used for the whole experiment was distilled water. The activity and appearance of the animals were monitored before and after the experiment 0.5 h (±5 min), 1 h (±5 min), 2 h (±5 min), 4 h (±10 min), and 6 h (+/15 min) in daily and continuously to 14 days, respectively.

### 2.4. Animals and Experimental Design

The 8-week db/db (BKS.Cg-Dock7^m^ +/+ Lepr^db^/J) male mice (30–40 g, fasting blood glucose above 11.1 mmol/l), db/dm mice (for the normal control group, 20–25 g), and all experiment procedures (including study protocol) were approved by the Changzhou Cavens Lab Animal Co., Ltd (Changzhou Cavens Ethics Committee on Animal Experimentation, China, production certificate no: SCXK2016-0010). All mice were maintained under a specific pathogen-free (SPF) condition with 22–24°C temperature, 45–80% humidity, 150–300lx light for 12 hours alternating between day and night. The normal control group (NC) is the nondiabetic mice (db/dm). The db/db male mice were divided into five groups: diabetic control (DC), diabetic with metformin (DMF, 200 mg/kg *bw*), diabetic with low dose KWZ (DKL, 40 mg/kg *bw*), diabetic with medium-dose KWZ (DKM, 80 mg/kg *bw*), and diabetic with high dose KWZ (DKH, 120 mg/kg *bw*), and each group consisted of eight mice, fed with a regular chow diet. The KWZ was dissolved and diluted in water and then delivered by oral gavage daily for 4 weeks. The NC and DC groups received an equal volume of vehicles (saline). Bodyweight and fasting blood glucose (FBG) levels in each group of mice were monitored weekly. The oral glucose test (OGTT) and insulin tolerance test (ITT) were carried out at week 4. After 4 weeks of administration, all mice were sacrificed, and then the plasma samples and liver were collected. The collected samples were immediately stored at −80°C for further analysis.

### 2.5. Oral Glucose Tolerance and Insulin Tolerance Tests

OGTT and ITT were conducted at the end of the administration. After fasting for 6 hours, OGTT was performed by given of glucose 2 g/kg. Blood glucose was measured at 0, 15, 30, 60, 90, and 120 min.

ITT was carried out at 36 h before the OGTT. After fasting for 6 hours, all animals were injected with insulin at 0.75 U/kg intraperitoneally. Orbital blood was taken before and 15, 30 60, 90, and 120 min, respectively, and insulin content was measured.

All experimental data were evaluated by calculating the area under the blood glucose curve (AUC). AUC (mmol/L. h) = (*X*_0_ + *X*_15_) × 0.25/2 + (*X*_15_ + *X*_30_) × 0.25/2 + (*X*_30_ + *X*_60_) × 0.5/2 + (*X*_60_ + *X*_90_) × 0.5/2 + (*X*_90_ + *X*_120_) × 0.5/2.

*X*_0_, *X*_15_, *X*_30_, *X*_60_, *X*_90_ and *X*_120_ represent the blood glucose and Insulin sensitivity values before and after the administration at 15, 30, 60, 90, and 120 min, respectively.

### 2.6. Lipid Profiles in Serum and Liver

The serum and liver tissues were collected after the animal sacrifice, and the levels of LDL-C, HDL-C, FFA, TC, TG, and serum Fructosamine were measured using assay kits according to the manufacturer's instructions.

### 2.7. Measurement of Oxidative Stress Markers in Liver

At first, the liver tissues of mice in each group were homogenized and centrifuged with Phosphate-buffered saline (PBS). The protein content was measured in the collected supernatant using Thermo Science Pierce BCA Protein Assay Kit (Rockford, USA). Supernatant aliquots were used to determine the liver oxidative stress markers, including HK, CAT, T-SOD, GSH-PX, and MDA were purchased from Nanjing Jiancheng Bioengineering Institute (China). The test procedure supplied with each commercial kit was followed.

### 2.8. Western Blotting

The total protein of the liver samples was extracted with RIPA lysis buffer (150 mM NaCl, 1% NP-40, 0.5% DOC, 0.1% SDS, 50 mM Tris (pH7.3), 1 mM PMSF and 1 × PI). Protein concentration was measured by using Thermo Science Pierce BCA Protein Assay Kit (Rockford, USA). Heat a 20 *μ*l sample with 5x loading buffer to 95–100°C for 5 min; cool on ice. The protein (100 *μ*g) samples were separated by SDS-PAGE on 10% polyacrylamide gels gel (10 cm × 10 cm) for 120 min at 110 volts and then transferred to PVDF membranes. After transfer, wash nitrocellulose membrane with 25 ml TBS for 5 min at room temperature. Then, there is incubation with blocking buffer (1X TBST with 5% w/v nonfat dry milk) for 1 hour at room temperature; after that, the membranes were blotted with primary antibodies (1 : 1000) overnight at 4°C. After being washed three times with TBST, the membranes were incubated by a horseradish peroxidase-conjugated secondary antibody. Visualization was detected with ECL Western Blotting Detection Reagent from GE Healthcare.

## 3. Results

### 3.1. Safety Evaluation of KWZ in the KM Mice

KWZ toxicity was tested using KM mice in the preliminary experiments. The test results including the maximum dose of KWZ (4500 mg/kg), bodyweight changes, and rate of death are shown in Table 1. According to the result of the experiment, 4500 mg/kg as a maximum dose used in this experiment was safe for animals.

### 3.2. Effect of KWZ on Bodyweight and FBG in the Diabetic db/db Mice

**Figure 1:**
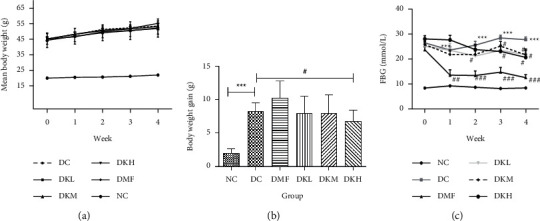
Effects of KWZ on body weight, weekly body weight gain, and FBG. (a) Weekly body weight changes. (b) Weekly body weight gain during the treatment in each group. (c) FBG levels. Data are expressed as mean ± SEM of 8 mice in each group. Data were analyzed by one-way ANOVA followed by a post hoc Tukey test. ^∗∗∗^*P* < 0.001 compared with NC. ^#^*P* < 0.05, ^##^*P* < 0.01 and ^#^*P* < 0.001 compared with DC. NC, normal control; DC, diabetic control; DMF, diabetic metformin; DKL, diabetic KWZ low dose; DKM, diabetic KWZ medium dose; DKH, diabetic KWZ high dose.

The weekly bodyweight and mean bodyweight gain were shown in Figures 1(a) and 1(b).  Figure 1(a) expressed the changes in bodyweight during the treatment in each group (before and after the treatment). Throughout the experiment, the weight of the mice in each group was increased, and differences were not significant. The bodyweight of mice was increased at the following groups: NC: 10.04%, DC: 18.06%, DMF: 22.81%, DKL: 18.06%, DKM: 17.58%, DKH: 14.98%. Compared with the NC group, the diabetic control group's bodyweight gain was significantly increased (*P* < 0.001). DKL, DKM, and DKH groups were significantly different from the DC group, indicating that KWZ maintains the stability of weekly body weight compared to the DMF group.

The weekly FBG levels of diabetic db/db mice were shown in Figure 1(c), and the weekly FBG levels of the DC group (*P* < 0.001) were significantly higher than the NC group. Compared with the DC group, the FBG level dropped from the second week to the fourth week, especially in the DKM group (*P* < 0.05). At the end of the experiment, KWZ significantly reduces the FBG level of DKL (*P* < 0.05), DKM (*P* < 0.05), and DKH (*P* < 0.05) groups.

### 3.3. Effects of KWZ on OGTT and ITT in the Diabetic db/db Mice

**Figure 2:**
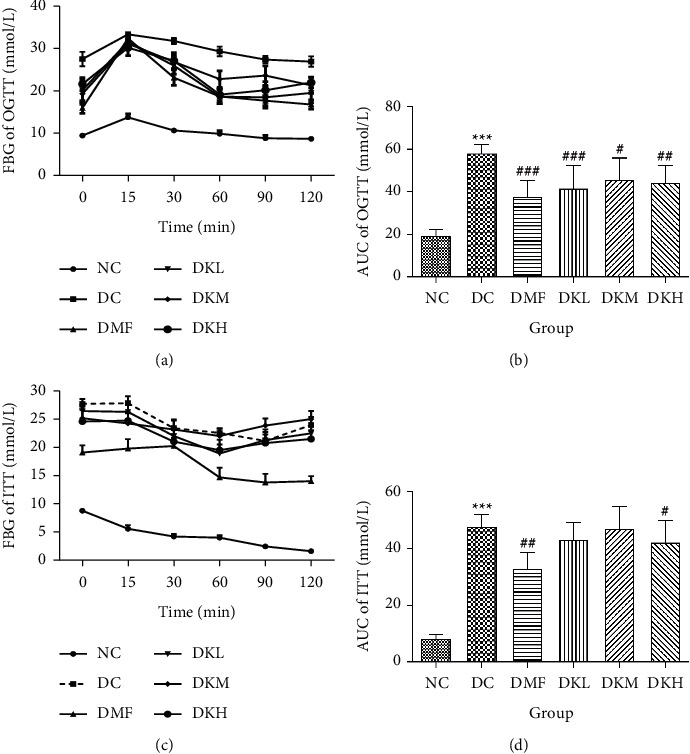
Effects of KWZ on OGTT, and ITT levels in diabetic db/db mice. (a) Results of OGTT. (b) AUC of OGTT in each group. (c) Results of ITT. (d) AUC of OGTT in each group. Data are expressed as mean ± SEM of 8 mice in each group. The method of data analysis, comparison, and expression between groups were the same as in Figure 1.

The OGTT and ITT were conducted at the end of the experiment. The results are illustrated in Figure 2. The blood glucose concentration at all time points during the OGTT and AUC was significantly higher in the DC group compared with the NC group. As shown in Figures 2(a) and 2(b), the OGTT levels at 15–60 min in the DKL (*P* < 0.001), DKM (*P* < 0.05), and DKH (*P* < 0.01) groups were significantly decreased compared to the DC group. Significant differences existed in blood glucose at 90 and 120 min between the DKL (*P* < 0.001) and DKH (*P* < 0.01) groups. AUC for blood glucose decreased significantly at 0–120 min in the DKL, DKM, and DKH groups compared with the DC group, but dose-dependent manner data were not analyzed.

Figures 2(c) and 2(d) showed that the blood glucose at all time points during the ITT and AUC levels of the diabetic db/db mice in the DKH (*P* < 0.05) and DMF (*P* < 0.01) was significantly lower than that of the DC group. Compared with the DC group, the ITT level at 15–60 min in DKH (*P* < 0.05) group was significantly decreased. No significant difference in the three doses of KWZ was observed at other points in time.

### 3.4. Effects of KWZ on Lipid Profiles and FAA in Serum/Liver of Diabetic db/db Mice

**Figure 3:**
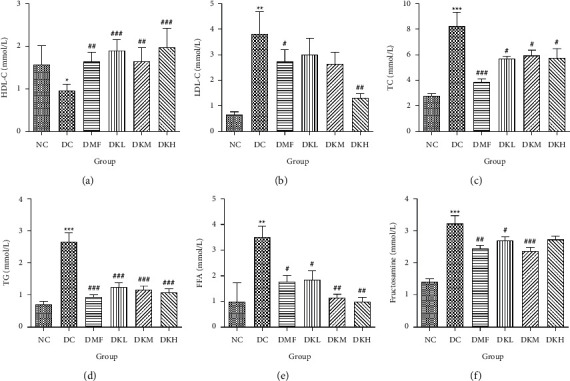
Effects of KWZ on serum lipid levels in diabetic db/db mice. (a–f) represent the levels of HDL-C, LDL-C, TC, TG, FFA, and Fructosamine. Data are expressed as mean ± SEM of 8 mice in each group. The methods of data analysis, comparison, and expression between groups were the same as in Figure 1.

As shown in Figure 3, the DC group had significantly decreased the serum levels of HDL-C and increased the serum levels of LDL-C, TC, TG, FFA, and fructosamine compared to the NC group. After four weeks of treatment, KWZ significantly increased the HDL-C level in DKL (*P* < 0.001), DKH (*P* < 0.01), and DKH (*P* < 0.001) group (Figure 3(a)) and decreased the LDL-C levels in DKH group (Figure 3(b), *P* < 0.01) compared to the DC group. DKL, DKM, and DKH groups also showed a similar effect on decreasing serum TG (Figure 3(c), *P* < 0.05), TC (Figure 3(d), *P* < 0.001), FFA (Figure 3(e), *P* < 0.05 or *P* < 0.01), and fructosamine levels (Figure 3(f), *P* < 0.05 or *P* < 0.001). However, the DKL and DKM groups in LDL-C levels and then the DKH group in fructosamine levels did not show a significant effect.

**Figure 4:**
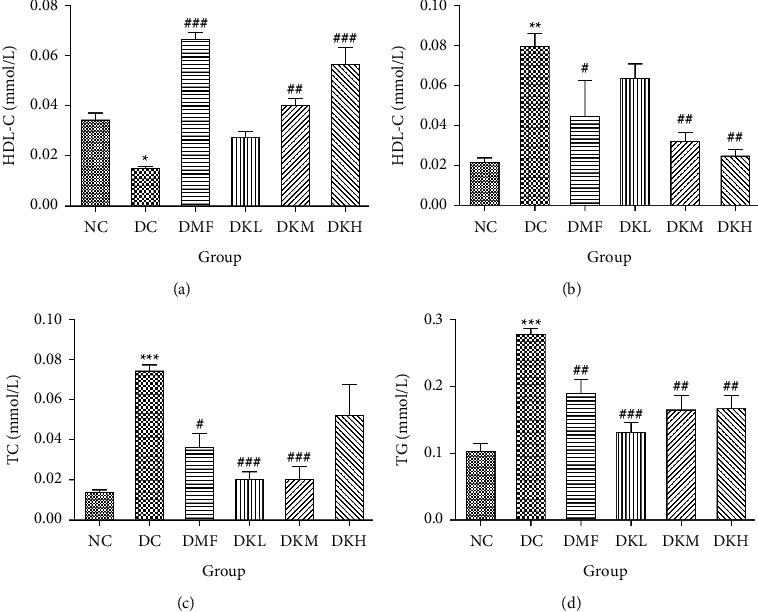
Effects of KWZ on liver lipid levels in diabetic db/db mice. (a–d) Levels of HDL-C, LDL-C, TC, and TG. Data are expressed as mean ± SEM of 8 mice in each group. The methods of data analysis, comparison and expression between groups were the same as Figure 1.

The liver levels of HDL-C, LDL-C, TC, and TG were shown in Figure 4. Compared to the NC group, the DC group had shown a significant difference in those parameters. The DMF (*P* < 0.001), DKM (*P* < 0.01), and DKH (*P* < 0.01) groups showed a significant increase in HDL-C levels (Figure 4(a)). Compared to the DC group, the DMF (*P* < 0.05), DKM (*P* < 0.01), and DKH (*P* < 0.01) groups decreased the LDL-C (Figure 4(b)) levels in diabetic mice's liver. Moreover, KWZ significantly improved the TC and TG levels compared to the DC group. The DMF (*P* < 0.05), DKL (*P* < 0.001), DKM (*P* < 0.001), and DKH (*P* < 0.01) significantly reduced the TC and TG levels, respectively, and the effect of DKL in TC and TG is mostly similar to that of the NC group. These results suggest that KWZ had a beneficial effect on regulating the serum and liver lipid levels and serum FFA levels.

### 3.5. Effects of KWZ on Enzymic Antioxidants and Nonenzymic Antioxidants Status in the Livers of Diabetic db/db Mice

Table 2 shows the activities of HK, glycogen, T-SOD, CAT, MDA, and GSH-PX in normal and diabetic db/db mice's liver. The results showed that DC group mice exhibited the increased activities of MDA level (*P* < 0.05) and a significant decrease in the activities of HK (*P* < 0.05), glycogen (*P* < 0.01), T-SOD (*P* < 0.05), CAT (*P* < 0.01), and GSH-PX (*P* < 0.05) levels when compared with the NC group mice. Conversely, the KWZ treatment groups such as DKL, DKM, DKH, and DMF significantly increased the HK, glycogen, T-SOD, CAT, MDA, and GSH-PX levels compared to the DC group. The MDA levels were reduced by KWZ treatment in DKL (*P* < 0.05), DKM (*P* < 0.05), and DKH (*P* < 0.05) groups. It was easy to observe from the results that KWZ treatment significantly improved the levels of enzymic antioxidants and nonenzymic antioxidants in diabetic db/db mice and was close to that of normal.

Data are expressed as mean ± SEM of 8 mice in each group, The methods of data analysis, comparison, and expression between groups were the same as Figure 1.

### 3.6. Effect of KWZ on the ER Stress in the Diabetic db/db Mice's Livers Tissue

**Figure 5:**
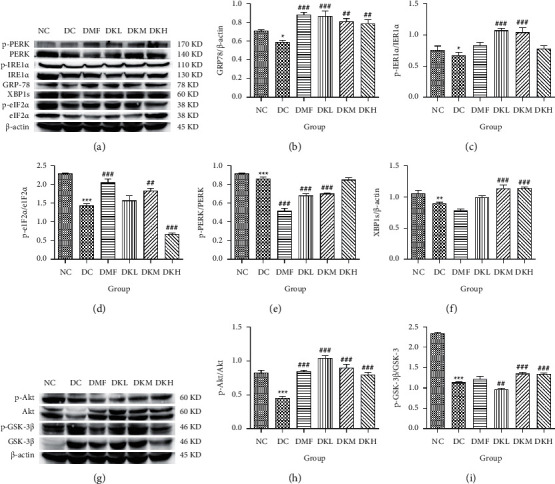
Effects of KWZ treatment on ER stress in the livers of diabetic db/db mice. (a–f) Representative western blots for total protein and phosphorylation expression of GRP78, p-IRE1*α*/IRE1*α*, p-eIF2*α*/eIF2*α*, p-PERK/PERK, and XBP1s in the livers of diabetic db/db mice. (g–i) p-Akt, Akt, p-GSK-3*β*, GSK-3*β* molecules expression. Data are expressed as mean ± SEM of 8 mice in each group. The methods of data analysis, comparison, and expression between groups were the same as in Figure 1.

The phosphorylation and dephosphorylation levels of IER1*α*, PERK, e1F2*α*, and XBP1s and GRP78 in diabetic db/db mice by western blot analysis are illustrated in Figures 5(a)–5(f) (original western blot images of IER1*α*, PERK, e1F2*α*, XBP1s, and GRP78 are shown in supplementary material (available here)). The protein expressions of p-eIF2*α*/eIF2*α* (*P* < 0.001), p-IRE1*α*/IRE1*α* (*P* < 0.05), and XBP1s (*P* < 0.01) in the DC group were significantly reduced compared to the NC group. Figures 5(b)–5(f) illustrated that the expression levels of GRP78 and XBP1s in KWZ treatment groups were significantly increased and improved the phosphorylation of eIF2*α*, IRE1*α*, and PERK compared to the DC group in the diabetic db/db mice's liver. Also, the results of protein expression showed a significant statistical difference between the model group and each treatment group.

Additionally, the all dosage of KWZ was found to significantly increase in the expression of p-Akt/Akt (Figures 5(g) and 5(h)) and p-GSK-3*β*/GSK-3*β* (Figure 5(g) and 5(i)) in a dose-dependent manner in the livers of diabetic db/db mice compared to the DC group (original western blot images of Akt and GSK-3*β* are shown in supplementary material (available here)). These data results indicate that KWZ treatment may improve the stability of ER, preventing the damage of insulin Beta cells and alleviating insulin resistance.

## 4. Discussion

Results of our study demonstrated that ER stress was associated with hyperglycemia or insulin resistance in vitro, suggesting that ER stress was involved in the pathogenesis of T2DM. It has been reported that diabetes mellitus and its complications lead to hepatic ER stress and inflammation, which can induce the development of insulin resistance in several tissues [23, 24]. The inhibition of ER stress by KWZ ameliorated insulin resistance, reduced FBG, restored biochemical indicators, and improved the glucose metabolism and lipid profiles in diabetic db/db mice.

OGTT and ITT are also the main indicators of insulin resistance [25]. The results of our study showed that KWZ improved the OGTT and ITT levels at 15–30 min in diabetic mice. Lipid metabolisms can directly respond to the changes in blood glucose in T2DM [26]. The disorder of the lipid metabolisms leads to dyslipidemia and induces excessive FFA, which increased the risk of insulin resistance [27]. The increase in TC and TG deposition contributes to fatty liver and liver injury. Fructosamines are glycated serum proteins that depend on their life span and reflect glycemic control over the previous 2 to 3 weeks [28]. The previous study showed that the increased serum levels of TG, TC, and LDL-C and decreased serum levels of HDL-C were associated with a pronounced increase in cardiovascular risk indices of diabetic rats [29, 30]. In our study, KWZ has shown desirable effects on regulating lipid metabolism. Particularly, it can successfully increase the serum and liver HDL-C levels and reduce the LDL-C, TC, and TG levels. Serum levels of FFA and Fructosamines were also significantly reduced by KWZ.

The liver is one of the most important secretory organs in the body, a major source of glucose production, and it became the important investigation target of glucose lipid metabolism [31]. Glucose metabolism is determined largely by the glucose concentration in the portal vein, and then, liver enzymes that have a major role in the control of glucose metabolism [32]. Glycogen, as a glucose metabolism enzyme, is one of the key sources of blood glucose in the early stage of fasting [33]. KWZ treatment significantly increased the hepatic glycogen content in diabetic mice. HK is the Beta cell glucose sensor and is involved in the control of blood glucose [34]. Then, our results demonstrated that KWZ successfully induced the HK, which is consistent with the increase of insulin signaling activity. KWZ is rich in antioxidant components, and the previous study gave evidence of the antioxidant activity of the effective part [21]. Diabetes is related to oxidative stress, marked by increased production of free radicals or impaired antioxidant defenses [35, 36]. The antioxidant enzyme (SOD, CAT, and GSH-PX) and index of oxidative stress (MDA) were examined and significantly improved by KWZ treatment in the diabetic mice liver.

Under physiological conditions, the ER can process the unfolded or misfolded proteins and maintain the steady-state of the cell. The interruption of the ER homeostasis activates the ER stress response, also known as the unfolded protein response, and these responses are also caused by pathological conditions, such as expression of mutant misfolding proteins, as well as insufficient ER chaperone levels or Ca^2+^ content, changes in redox or ATP status, ER phospholipid depletion, and cholesterol accumulation, and these conditions are always involved in the pathogenesis of diabetics and its complications [37]. Several mechanisms, such as PERK, IRE1, and ATF6 pathways, can alleviate ER stress and restore ER to its normal physiological conditions. These pathways are also critical in chronic metabolic diseases such as obesity, insulin resistance, and T2DM. When we reviewed the ER stress response, a lot of research articles on the treatment of drugs in T2DM db/db mice have been found, and most of them focus on the mechanism of ER stress response in diabetic nephropathy (DN) [38, 39], but researches about endoplasmic reticulum in glucose metabolism and insulin sensitivity have been largely ignored [23, 40]. In this study, we found that the natural drug KWZ successfully activated ER chaperone GRP78 and regulated the abnormal expression of proteins related to the ER stress response. GRP78 is an ER chaperone binding with PERK, IRE1, and ATF6 under nonstressed conditions. In response to the ER stress, GRP78 dissociated from the PERK, IRE1, and ATF6 leads to their activation. During the hepatic ER stress, PERK phosphorylates eIF2a leading to attenuation of translation and induction of ATF4, and relieving the ER workload [5], eIF2a can also indirectly hamper the insulin signaling through serine phosphorylation of IRS1 or some inflammatory factors, such as JNK [41], and then, it causes unconventional splicing of XBP1 mRNA and translation into the transcription factor XBP1s [42]. XBP1 nuclear translocation is increased by insulin signaling, through the binding of the PI3K regulatory subunit p85 to XBP1, and then controls lipogenic enzyme transcription by a still unknown mechanism [43]. One previous study showed that ursodeoxycholic acid remarkably induced the upregulation of ER stress-related proteins in HG-induced podocyte apoptosis and db/db mice, including GRP78, PERK, ATF6, IRE1, XBP-1s, and active caspase-12 occurred, and our results were similar to those of these investigators [44]. After treatment with KWZ, significant activations were observed through the PERK/eIF2*α* and IRE1/XBP1 pathways, which were the results of GRP78 expression, indicating that KWZ might be beneficial for alleviating insulin resistance through the improving stability of the ER.

In conclusion, the results of this study indicated that the administration of KWZ reduced the blood glucose level, improved the glucose metabolism and lipid profiles, and restored the activities of biochemical indicators in diabetic mice. The western blot result showed that KWZ significantly regulated the ER stress response through the PERK/eIF2*α* and IRE1/XBP1 signaling pathways, which are involved in glucose metabolism and insulin resistance. The study provides a mechanism of KWZ action in the db/db T2DM male mice and may be considered as promising new drugs for the prevention and treatment of metabolic syndrome and T2DM.

## Figures and Tables

**Table 1:** The body weight changes of KM mice when the maximum dose was given.

Dose (g/kg. bw)	Body weight (day/g)					Number of dead animals (*n*)	Death rate (%)
	0	3	5	7	14		
0	41.68 ± 0.71	42.54 ± 0.72	42.92 ± 0.86	43.80 ± 0.57	44.32 ± 1.34	—	0.00
4500	41.45 ± 0.70	42.80 ± 0.86	44.29 ± 0.96	46.22 ± 0.80	47.21 ± 1.47	—	0.00

**Table 2:** Effects of KWZ on HK, glycogen, and antioxidant enzyme in experimental mice's liver with four-week treatment.

Groups	HK (U/g protein)	Glycogen (mg/g)	T-SOD (U/mg protein)	CAT (U/mg protein)	MDA (nmol/mg protein)	GSH-PX (U/mg protein)
NC	75.02 ± 56.07	25.50 ± 5.29	138.39 ± 9.29	313.52 ± 74.94	3.83 ± 0.73	1444.73 ± 119.24
DC	58.67 ± 16.20^*∗*^	9.13 ± 4.09^∗∗^	126.38 ± 11.71^*∗*^	195.06 ± 61.88^∗∗^	4.35 ± 0.98^*∗*^	1245.02 ± 111.71^∗∗^
DMF	81.13 ± 15.95^#^	27.23 ± 3.14^##^	146.89 ± 13.05^*∗*^	233.12 ± 43.10^*∗*^	2.92 ± 0.54^#^	1357.52 ± 132.67^#^
DKL	73.60 ± 16.76^#^	34.39 ± 16.32^##^	134.55 ± 16.31	347.54 ± 107.51^##^	2.55 ± 0.39^#^	847.55 ± 110.99
DKM	92.09 ± 26.34^##^	35.14 ± 13.03^##^	136.08 ± 14.71	352.42 ± 106.36^##^	2.39 ± 0.34^##^	1379.76 ± 117.72^##^
DKH	63.65 ± 12.29	38.25 ± 6.84^##^	140.53 ± 18.70^#^	336.20 ± 83.99^##^	2.62 ± 0.92^##^	1091.99 ± 155.77

## Data Availability

All the experimental data and results during the study are clearly shown in the manuscript.

## Supplementary Materials

Supplementary Materials Fig. S1. Expression of p-IRE1*α* Fig. S2. Expression of IRE1*α* Fig. S3. Expression of p-PERK Fig. S4. Expression of PERK Fig. S5. Expression of p-eIF2*α* Fig. S6. Expression of eIF2*α* Fig. S7. Expression of GRP78 Fig. S8. Expression of XBP1s Fig. S9. Expression of *ß*-actin Fig. S10. Expression of p-Akt Fig. S11. Expression of Akt Fig. S12. Expression of p-GSK-3*β* Fig. S13. Expression of GSK-3*β.*
