# Supplementary material for: Kursi Wufarikun Ziyabit Improves the Physiological Changes by Regulating Endoplasmic Reticulum Stress in the Type 2 Diabetes db/db Mice
Source: Evid Based Complement Alternat Med. 2021 Aug 16;2021:2100128. doi: 10.1155/2021/2100128 (PMC8384507; doi:10.1155/2021/2100128)
Supplement: Supplementary Materials — Fig. S1. Expression of p-IRE1α Fig. S2. Expression of IRE1α Fig. S3. Expression of p-PERK Fig. S4. Expression of PERK Fig. S5. Expression of p-eIF2α Fig. S6. Expression of eIF2α Fig. S7. Expression of GRP78 Fig. S8. Expression of XBP1s Fig. S9. Expression of ß-actin Fig. S10. Expression of p-Akt Fig. S11. Expression of Akt Fig. S12. Expression of p-GSK-3β Fig. S13. Expression of GSK-3β. [file 2100128.f1.pdf]

Supplementary Materials:

Kursi Wufarikun Ziyabit improves the physiological changes by regulating endoplasmic reticulum stress in the type 2 diabetes db/db mice

Salamet Edirs [a, b], Lan Jiang [a, b], XueLei Xin [a, b *], H. A. Aisa [a, b *]

[a] *The Key Laboratory of Plant Resources and Chemistry of Arid Zone, Xinjiang Technical Institute of Physics and Chemistry, Chinese Academy of Sciences, 40-1 Beijing Road, Urumqi, Xinjiang, 830011, China*

[b] *State Key Laboratory Basis of Xinjiang Indigenous Medicinal Plants Resource Utilization, Xinjiang Technical Institute of Physics and Chemistry, Chinese Academy of Sciences, 40-1 Beijing Road, Urumqi, Xinjiang, 830011, China*

Corresponding authors: Haji Akber Aisa*; XueLei Xin*

The Key Laboratory of Plant Resources and Chemistry of Arid Zone

Xinjiang Technical Institute of Physics and Chemistry

Chinese Academy of Sciences, Urumqi 830011, China

Email: haji@ms.xjb.ac.cn; Phone: 86-0991-3835679. Fax: 86-0991-3835679.

Email: xinxl@ms.xjb.ac.cn; Phone: 86-0991-3835679. Fax: 86-0991-3835679.

Contents:

Fig. S1. Expression of p-IRE1α

Fig. S2. Expression of IRE1α

Fig. S3. Expression of p-PERK

Fig. S4. Expression of PERK

Fig. S5. Expression of p-eIF2α

Fig. S6. Expression of eIF2α

Fig. S7. Expression of GRP78

Fig. S8. Expression of XBP1s

Fig. S9. Expression of β-actin

Fig. S10. Expression of p-Akt

Fig. S11. Expression of Akt

Fig. S12. Expression of p-GSK-3β

Fig. S13. Expression of GSK-3β

In all the figures: NC, normal control; DC, diabetic control; DMF, diabetic metformin; DKL, diabetic KWZ low dose; DKM, diabetic KWZ medium dose; DKH, diabetic KWZ high dose.

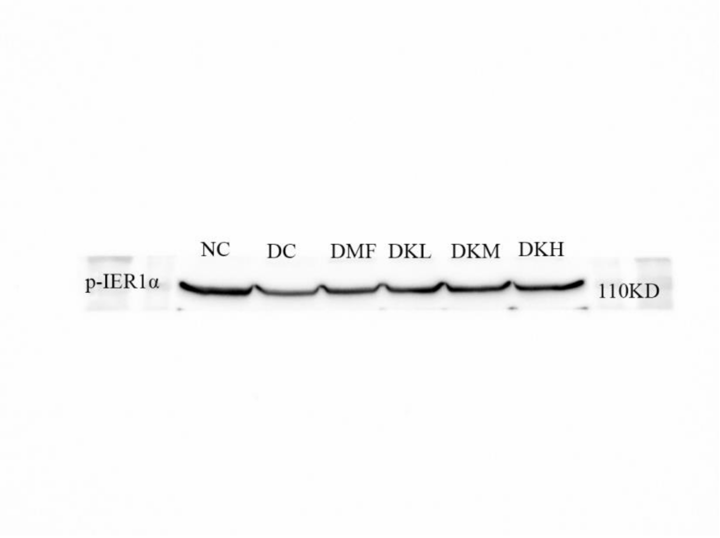

Fig. S1. Expression of p-IRE1α

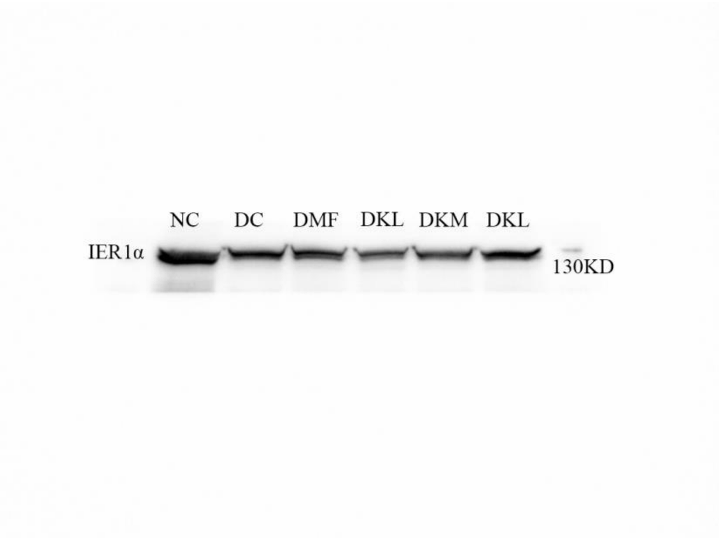

Fig. S2. Expression of IRE1α

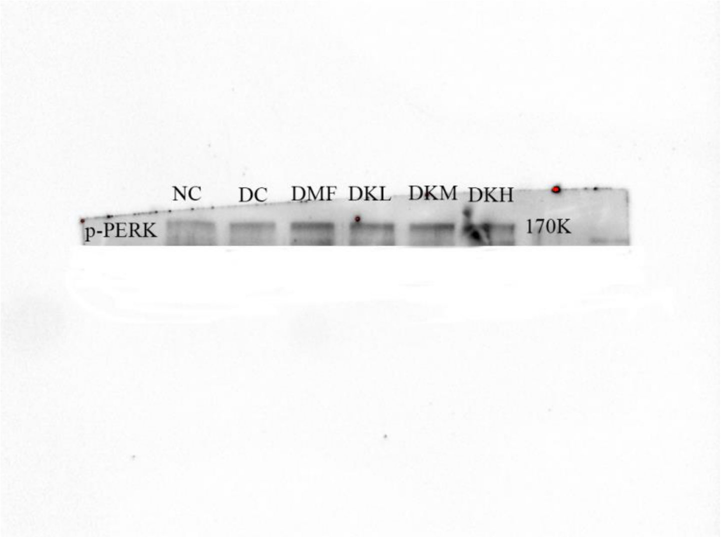

Fig. S3. Expression of p-PERK

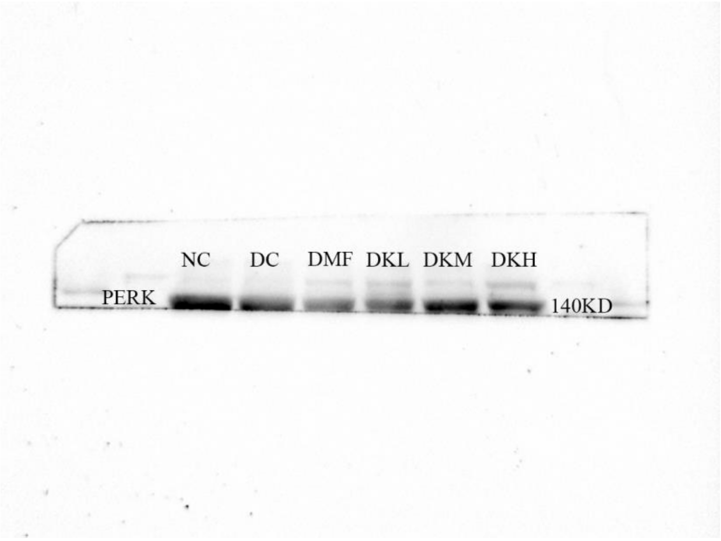

Fig. S4. Expression of PERK

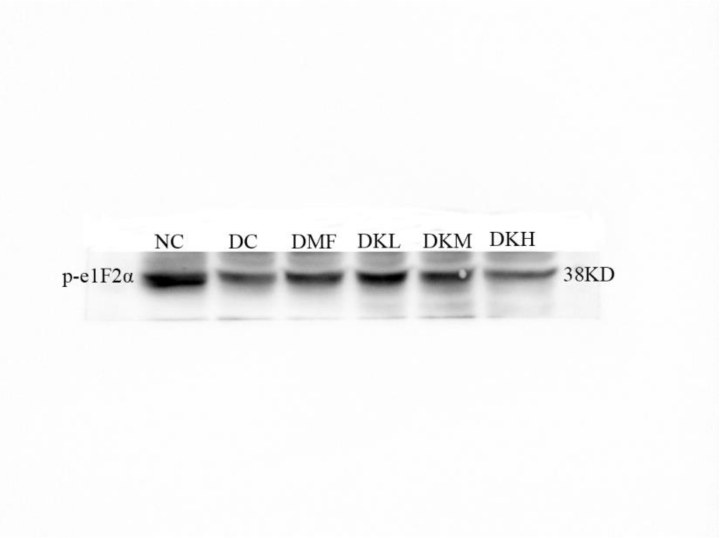

Fig. S5. Expression of p-eIF2α

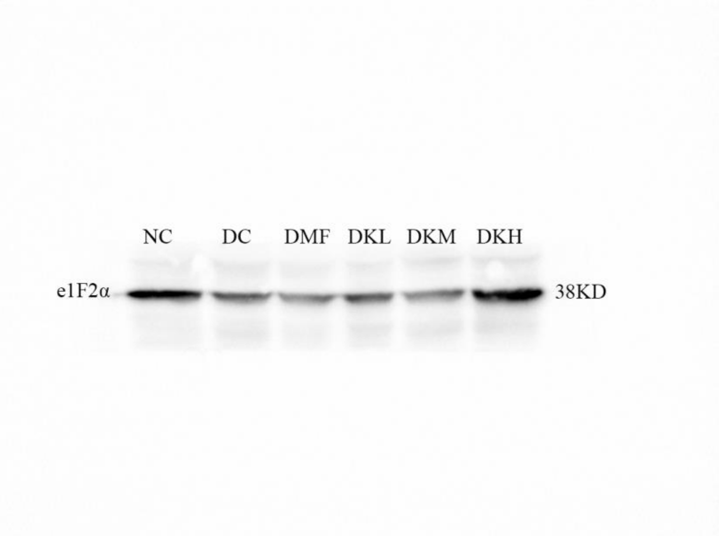

Fig. S6. Expression of eIF2α

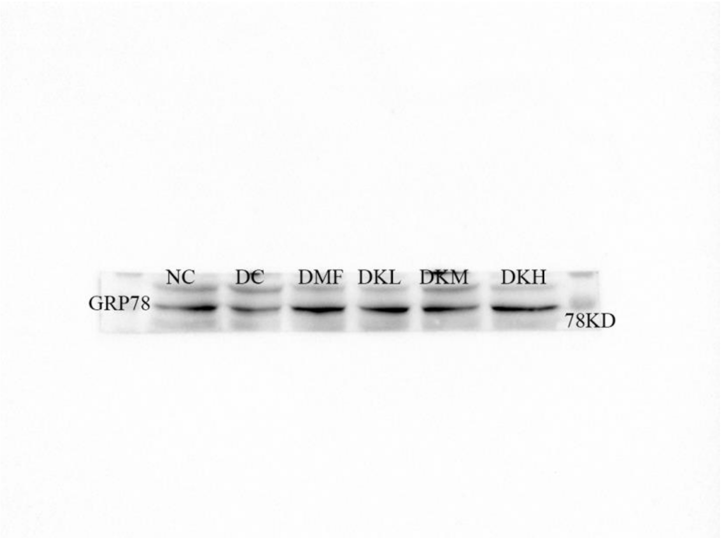

Fig. S7. Expression of GRP78

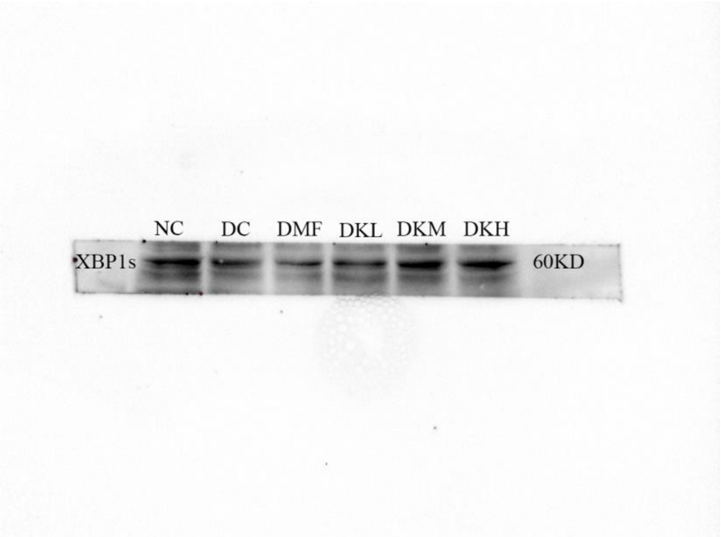

Fig. S8. Expression of XBP1s

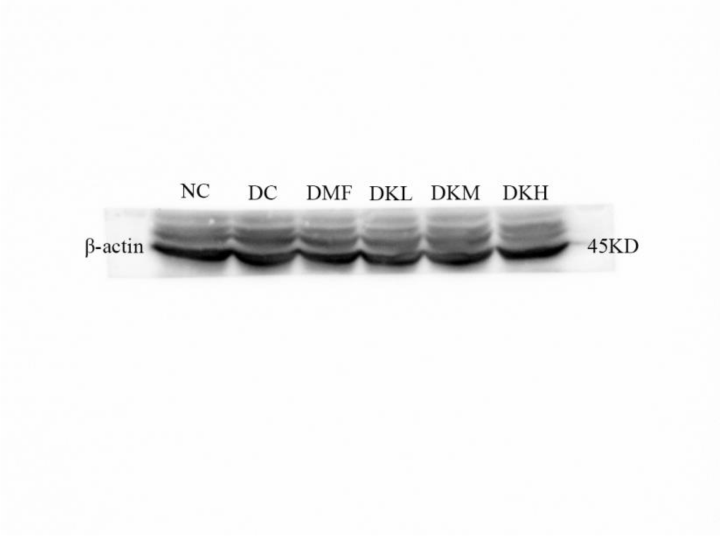

Fig. S9. Expression of β-actin

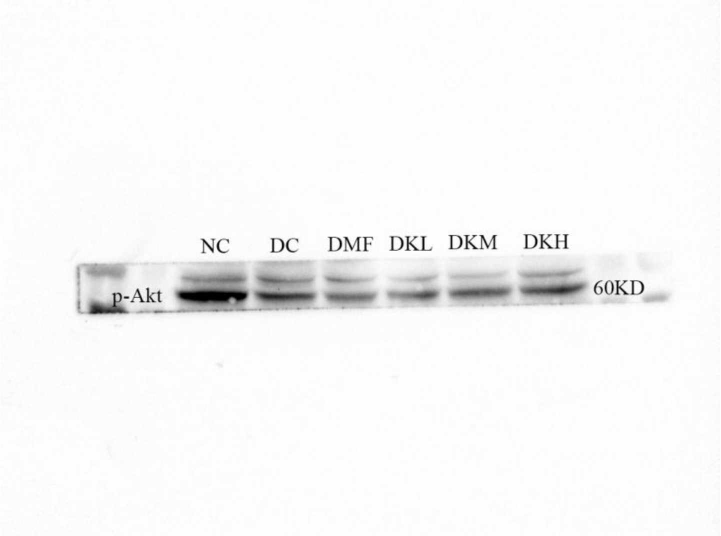

Fig. S10. Expression of p-Akt

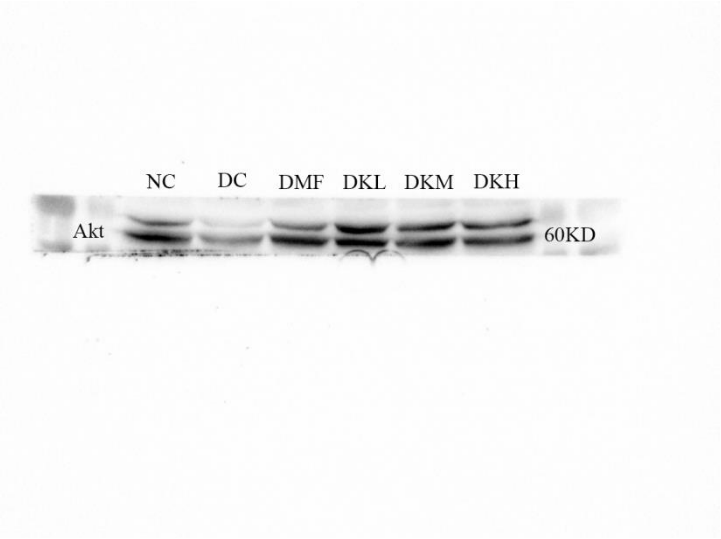

Fig. S11. Expression of Akt

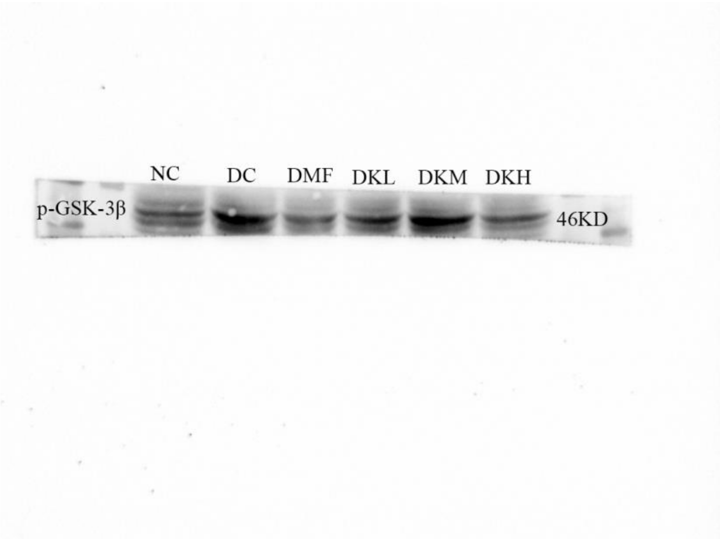

Fig. S12. Expression of p-GSK-3β

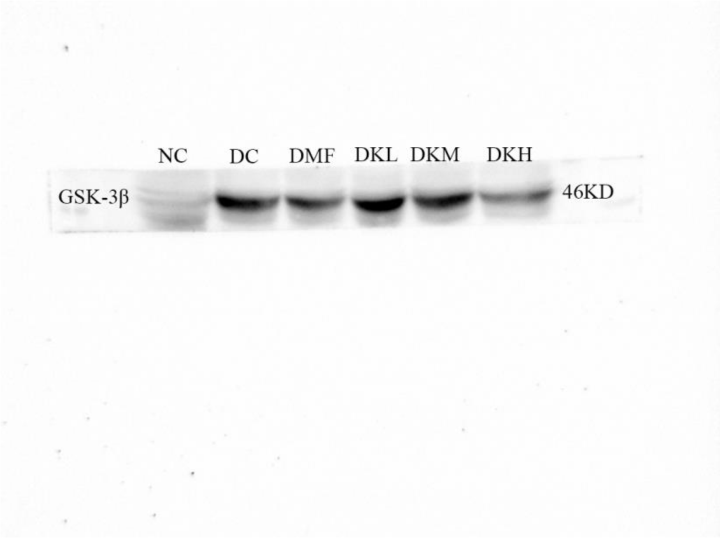

Fig. S13. Expression of GSK-3β
